# Supplementary material for: Cryo-EM structure and dynamics of the green-light absorbing proteorhodopsin
Source: Nat Commun. 2021 Jul 5;12:4107. doi: 10.1038/s41467-021-24429-6 (PMC8257665; doi:10.1038/s41467-021-24429-6)
Supplement: Supplementary file 1 — Supplementary Information [file 41467_2021_24429_MOESM1_ESM.pdf]

Supplementary Information:

Cryo-EM structure and dynamics of the green-light absorbing proteorhodopsin

Stephan Hirschi[1], David Kalbermatter[1], Zöhre Ucurum[1], Thomas Lemmin[2,3,*] & Dimitrios Fotiadis[1,*]

[1]Institute of Biochemistry and Molecular Medicine, University of Bern, 3012 Bern, Switzerland

[2]DS3Lab, System Group, Department of Computer Sciences, ETH Zurich, 8092 Zürich, Switzerland

[3]Trkola Group, Institute of Medical Virology, University of Zurich, 8057 Zürich, Switzerland

* Correspondence should be addressed to T.L. (email: thomas.lemmin@inf.ethz.ch) or D.F. (email: dimitrios.fotiadis@ibmm.unibe.ch)

Methods

Tag-free Purification of GPR

Cells expressing GPR were thawed and then disrupted by five cycles through a Microfluidizer (M-110P, Microfluidics) at 1500 bar. Membranes were isolated and washed twice in 50 mM Tris-HCl pH 8, 450 mM NaCl by repeated ultracentrifugation (1h at 150,000 g and 4°C) and homogenization. Finally, membranes were resuspended in purification buffer (20 mM Bis-Tris propane (BTP)-HCl pH 9, 50 mM NaCl, 10% (v/v) glycerol). Membranes corresponding to 3 L of bacterial cell culture were solubilized in 21 mL purification buffer containing 3% (w/v) Cymal-4 (Anatrace) on a turnover shaker overnight at 4°C. Solubilized membranes were ultracentrifuged (1h at 100,000 g and 4°C) to remove insoluble material. The remaining purification steps up to size exclusion chromatography (SEC) were performed at room temperature. GPR was first passed through 1 mL (bed volume) CM Sepharose (GE Healthcare) and then bound to 3 mL (bed volume) Q Sepharose (GE Healthcare), both equilibrated with purification buffer containing 0.8% (w/v) Cymal-4. The bound protein was washed by a two-step NaCl gradient using 20 mL and 40 mL of 20 mM BTP-HCl pH 9, 0.8% (w/v) Cymal-4 containing first 200 mM and then 275 mM NaCl. GPR was eluted using the same buffer containing 450 mM NaCl. The protein was concentrated using a Vivaspin 2 50 kDa concentrator (Sartorius) and further purified by SEC using a Superdex 200 Increase 10/300 column (GE Healthcare) equilibrated with 20 mM BTP-HCl pH 7.5, 150 mM NaCl, 0.8% (w/v) Cymal-4. Pentameric GPR fractions were pooled and concentrated to 3.5 mg/mL. Protein purity and homogeneity were assessed by SDS-PAGE and analytical SEC (Fig. S1).

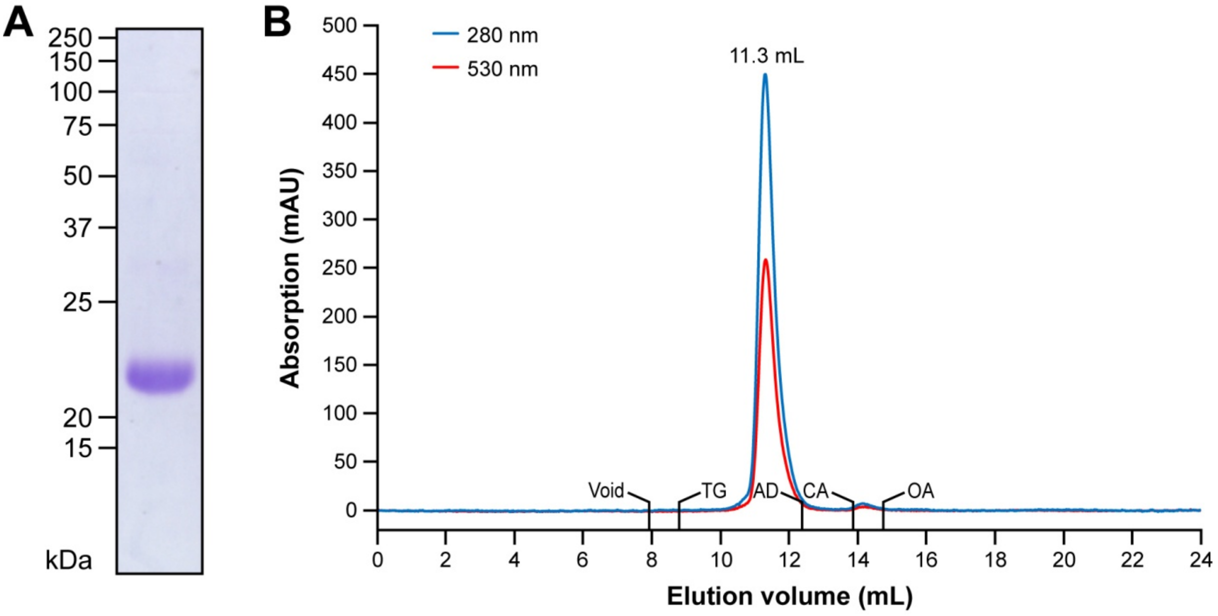

Fig. S1. Analysis of GPR purity and homogeneity. (A) Purified GPR runs as a single band at around 22 kDa on a 16% SDS-polyacrylamide gel. A representative gel is shown from three independent experiments with similar results. Source data are provided as a Source Data file. (B) Size exclusion chromatography profile of purified GPR on a Superdex 200 Increase 10/300 GL column (GE Healthcare). Absorption at 280 nm and 530 nm (retinal Schiff base absorption maximum) were recorded. GPR elutes at 11.3 mL as a single homogeneous peak, corresponding to a molecular weight of approximately 215 kDa for the ternary complex (protein, lipid and detergent). The elution volumes of the void and molecular weight marker proteins, including thyroglobulin (TG, 669 kDa), aldolase (AD, 158 kDa), conalbumin (CA, 75 kDa) and ovalbumin (OA, 43 kDa) are indicated.

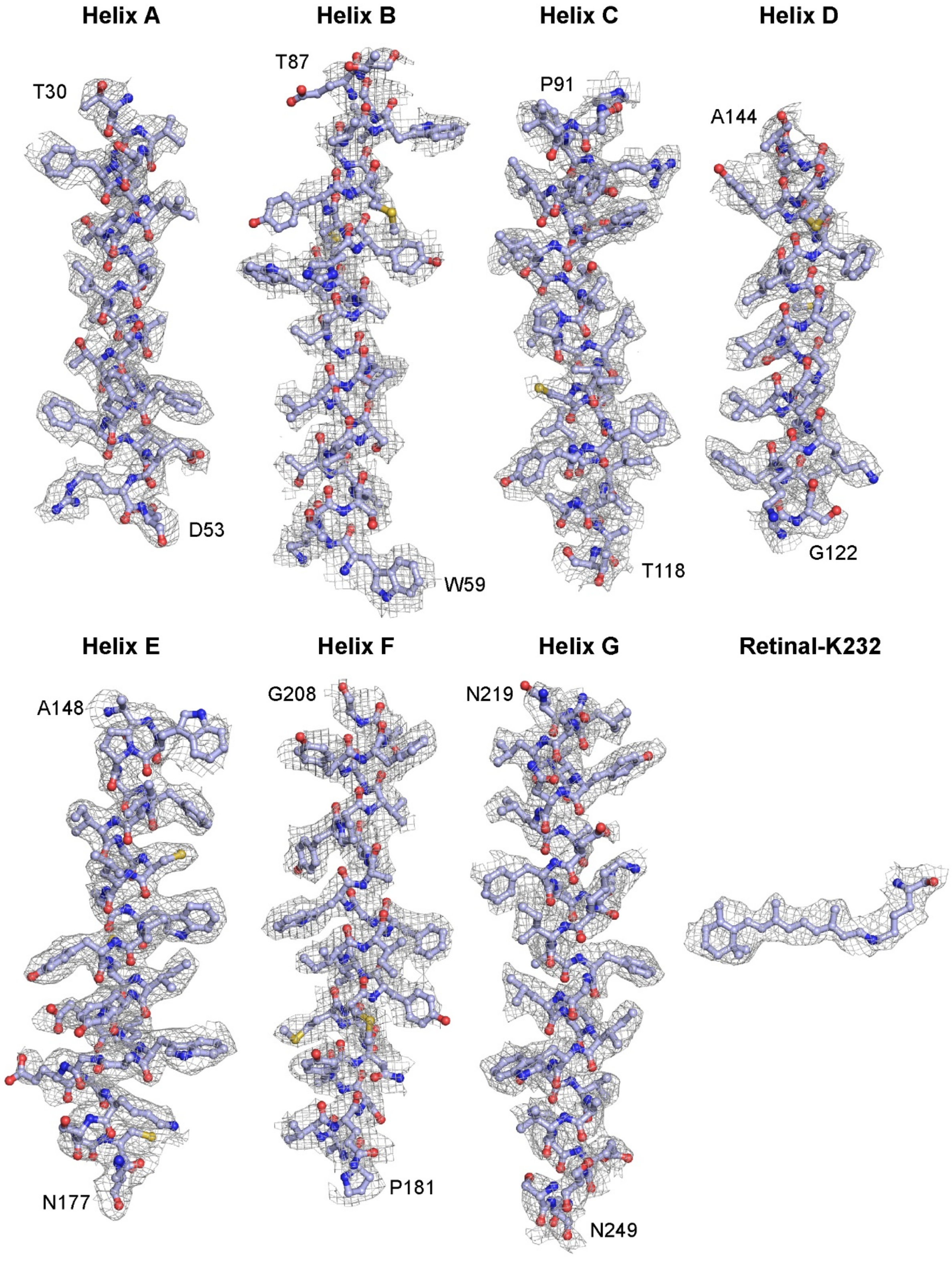

Fig. S2. Quality of the cryo-EM map. Amino acid residues of GPR transmembrane helices with corresponding densities are displayed, while loops are omitted. The first and last residues of each transmembrane helix are labelled. Additionally, the all-*trans* retinal chromophore is shown, which is covalently attached to K232 via a Schiff base.

A

GPR	100							
*Hot75*BPR	79.6	100						
*Med12*BPR	56.3	58.3	100					
ESR	35.8	34.1	34.9	100				
XR	30.5	26.9	24.7	31.0	100			
GR	29.0	26.0	25.5	28.3	50.9	100		
BR	26.0	26.9	27.3	27.2	24.4	25.1	100	
AR-1	22.1	23.5	23.0	27.4	23.8	24.5	57.4	100
	GPR	*Hot75*BPR	*Med12*BPR	ESR	XR	GR	BR	AR-1

B

```
● GPR (NMR)     1 M-KLLLILGSVIALPTFAAGGGDLDASDYTGVSFWLVTAALLASTVFFFV   49
                  | ||||||||||||||||||||||||||||||||||||||||||||||||
● GPR           1 MGKLLLILGSVIALPTFAAGGGDLDASDYTGVSFWLVTAALLASTVFFFV   50
                  ||||||||||.||||:|||.|||||.||..|||||||||.:||:||||||
● Hot75BPR      1 MGKLLLILGSAIALPSFAAAGGDLDISDTVGVSFWLVTAGMLAATVFFFV   50

● GPR          51 ERDRVSAKWKTSLTVSGLVTGIAFWHYMYMRGVWIETGDSPTVFRYIDWL  100
                  |||:||||||||||||||:||||||||:||||||:|||:|||||||||||
● Hot75BPR     51 ERDQVSAKWKTSLTVSGLITGIAFWHYLYMRGVWIDTGDTPTVFRYIDWL  100

● GPR         101 LTVPLLICEFYLILAAATNVAGSLFKKLLVGSLVMLVFGYMGEAGIMAAW  150
                  |||||.:.||||||||.|:||.|||||||.||||||..|:.||||:....
● Hot75BPR    101 LTVPLQVVEFYLILAACTSVAASLFKKLLAGSLVMLGAGFAGEAGLAPVL  150

● GPR         151 PAFIIGCLAWVYMIYELWAGEGKSACNTASPAVQSAYNTMMYIIIFGWAI  200
                  ||||||...|:||||||:.||||:|.:||||||.||||.||.||:.||||
● Hot75BPR    151 PAFIIGMAGWLYMIYELYMGEGKAAVSTASPAVNSAYNAMMMIIVVGWAI  200

● GPR         201 YPVGYFTGYLM-GDGGSALNLNLIYNLADFVNKILFGLIIWNVAVKESSN  249
                  ||.||..|||| |:|..|.|||||||||||||||||||||||||||||||
● Hot75BPR    201 YPAGYAAGYLMGGEGVYASNLNLIYNLADFVNKILFGLIIWNVAVKESSN  250

● GPR         250 A    250
                  |
● Hot75BPR    251 A    251
```

Fig. S3. Amino acid sequence comparison of microbial proton-pumping rhodopsins of known structure. (A) Sequence identity (%) between the full-length versions (i.e., including signal sequences) of green-light absorbing proteorhodopsin (GPR, UniProt accession number Q6J4G7), two blue-light absorbing proteorhodopsins (*Hot75*BPR, Q9AFF7 and *Med12*BPR, Q4PP54), *Exiguobacterium sibiricum* rhodopsin (ESR, B1YFV8), xanthorhodopsin (XR, Q2S2F8), *Gloeobacter violaceus* rhodopsin (GR, Q7NP59), bacteriorhodopsin (BR, P02945) and archaerhodopsin-1 (AR-1, P69052). Values were obtained using the Clustal Omega multiple sequence alignment algorithm (https://www.ebi.ac.uk/Tools/msa/clustalo/). (B) Global pairwise sequence alignment between GPR versions used to determine the NMR (red, Q9F7P4) and cryo-EM structure (green, Q6J4G7), and *Hot75*BPR (blue, Q9AFF7) employing the Needle algorithm (https://www.ebi.ac.uk/Tools/psa/emboss_needle/). The GPR construct used to determine the solution NMR structure is missing a glycine at position two, whereas BPR contains an additional glycine after M211, resulting in single gaps (highlighted in orange) compared to the GPR version used in this study. Only the first 50 amino acids of the alignment between the two GPR versions are shown since the remaining sequence is identical.

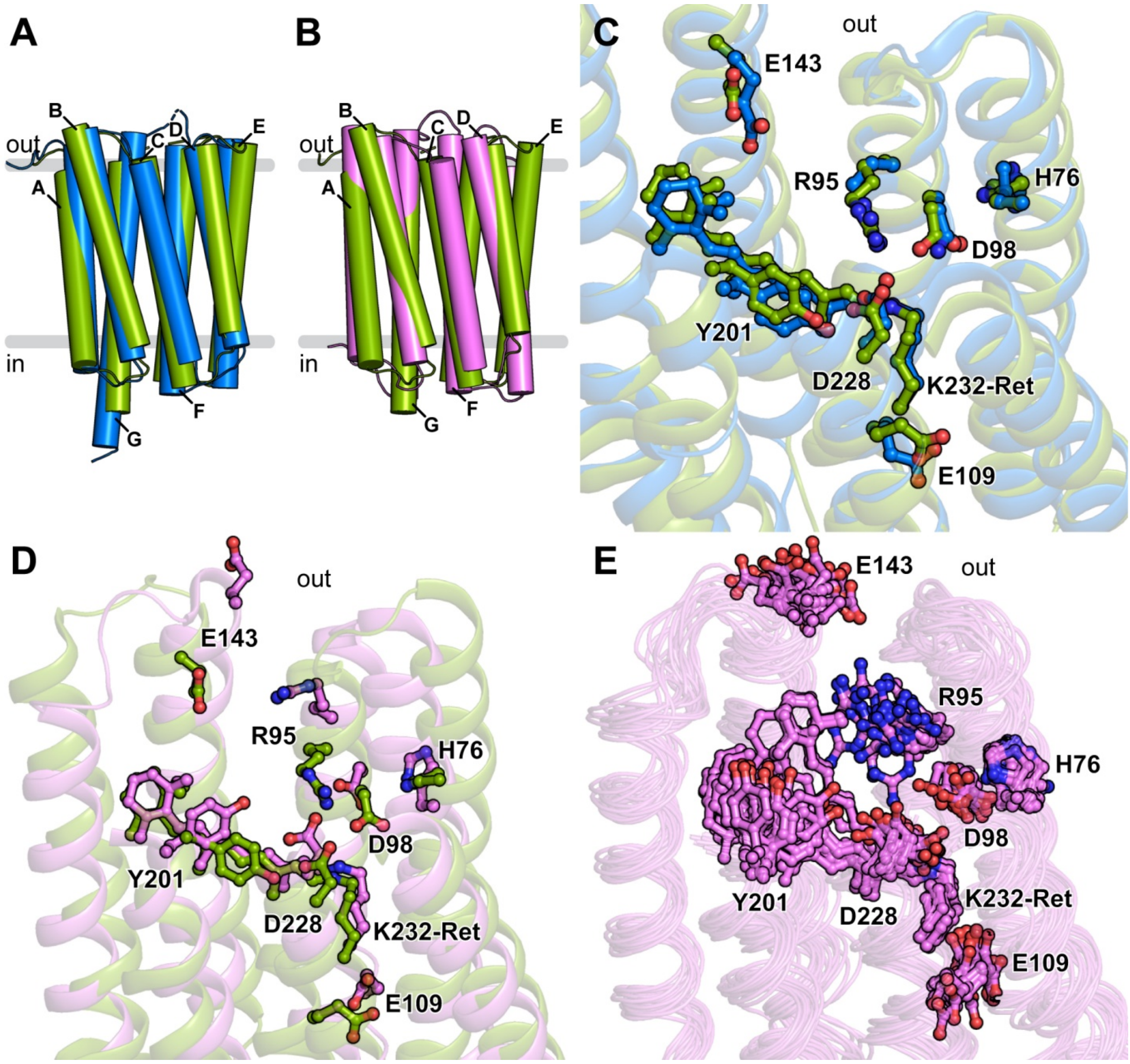

Fig. S4. Comparison of GPR and BPR structures. (A) Superposition of the GPR cryo-EM (green) and BPR (blue) structures. (B) Superposition of the GPR cryo-EM structure (green) and the best aligned GPR NMR structure (pink). (C) Comparison of GPR with the best aligned protomer of the BPR D97N (D98 in GPR) crystal structure (RMSD of 1.4 Å). (D) Comparison of GPR with the best aligned GPR NMR structure (RMSD of 4.0 Å). (E) Representation of the different models of the GPR solution NMR structure ensemble including the residues depicted in (D).

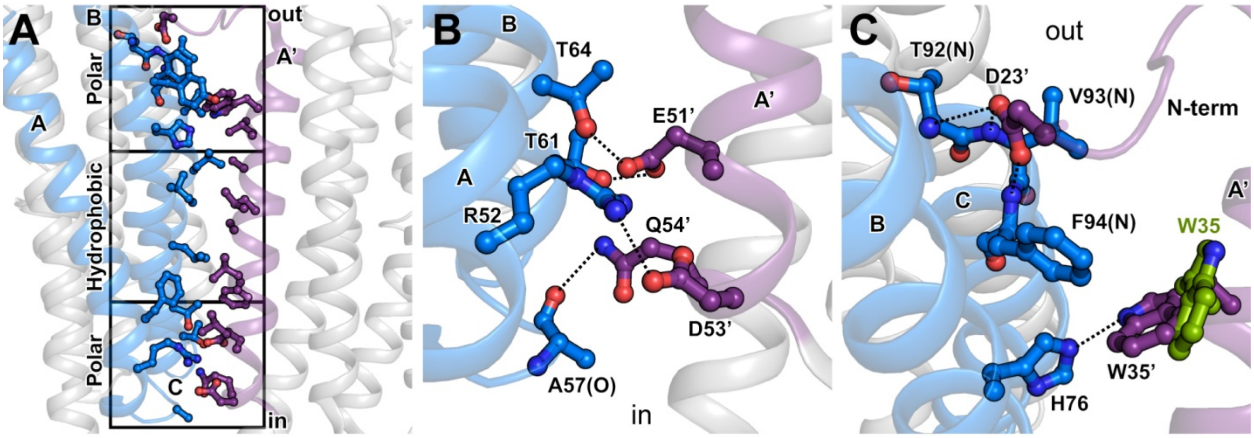

Fig. S5. Oligomerization interface between BPR protomers. (A) Different polar and hydrophobic interactions stabilize the oligomerization interface between BPR protomers (blue and purple). (B) Cluster of polar interactions at the intracellular solvent interface, comprising a number of hydrogen bonds and salt bridges between R52, A57, T61 and T64 of one protomer, and E51', D53' and Q54' of the adjacent protomer. (C) Hydrogen bonding network at the extracellular interface between the N-terminus (D23’) of one protomer (purple) and the backbone nitrogen atoms of T92, V93 and F94 of the second protomer (blue). In addition, the functional interaction between W35’ and H76 is shown, which is precluded due to the flipped-up position of W35 in GPR (green). Transmembrane helices participating in the interface are labelled.

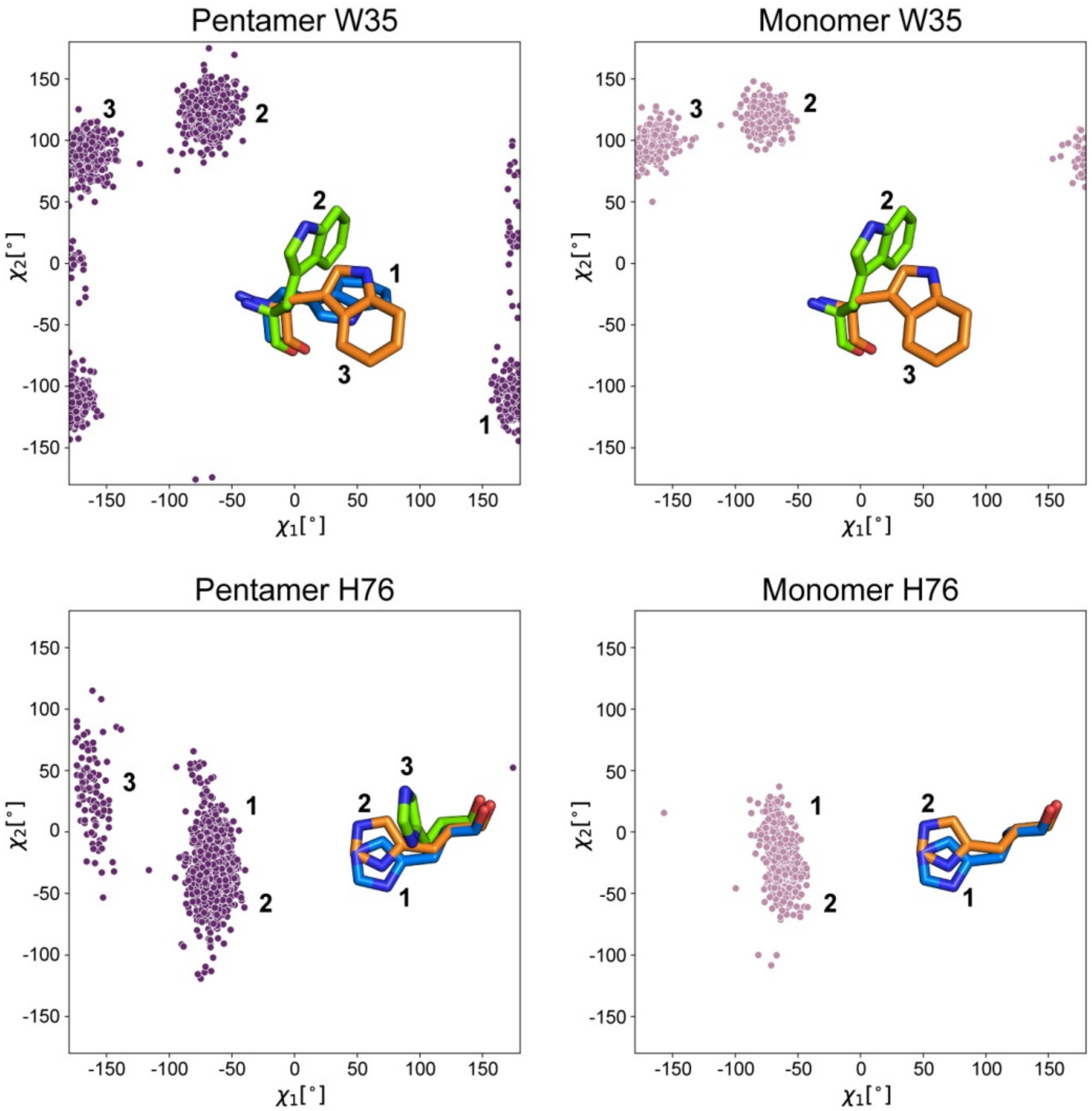

Fig. S6. Effect of GPR oligomerization on W35 and H76 rotamer conformations. The scatter plots indicate the χ_1 and χ_2 torsional angles for W35 and H76 during the MD simulations of pentameric and monomeric GPR. Each point represents a rotamer sampled during the simulation. Main clusters and corresponding conformations are numbered.

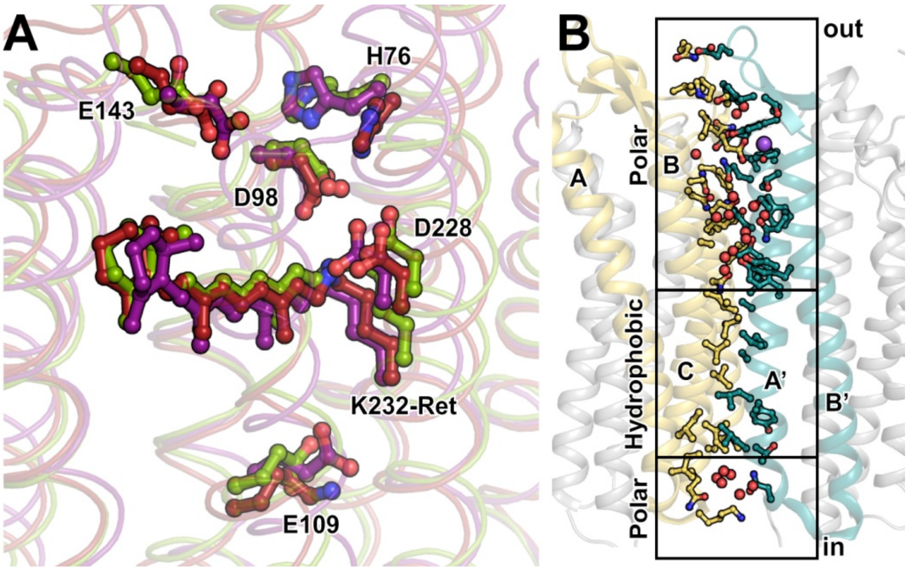

Fig. S7. Structural comparison of selected microbial rhodopsins. (A) Functional residues for the proton pumping activity of GPR (green), ESR (red) and GR (purple). Residue labelling corresponds to GPR. (B) Oligomerization interface of KR2 with different regions of polar and hydrophobic interactions indicated. Helices involved in forming the interface between protomers are highlighted in yellow and blue. A sodium ion is represented as a purple and water molecules as red spheres.

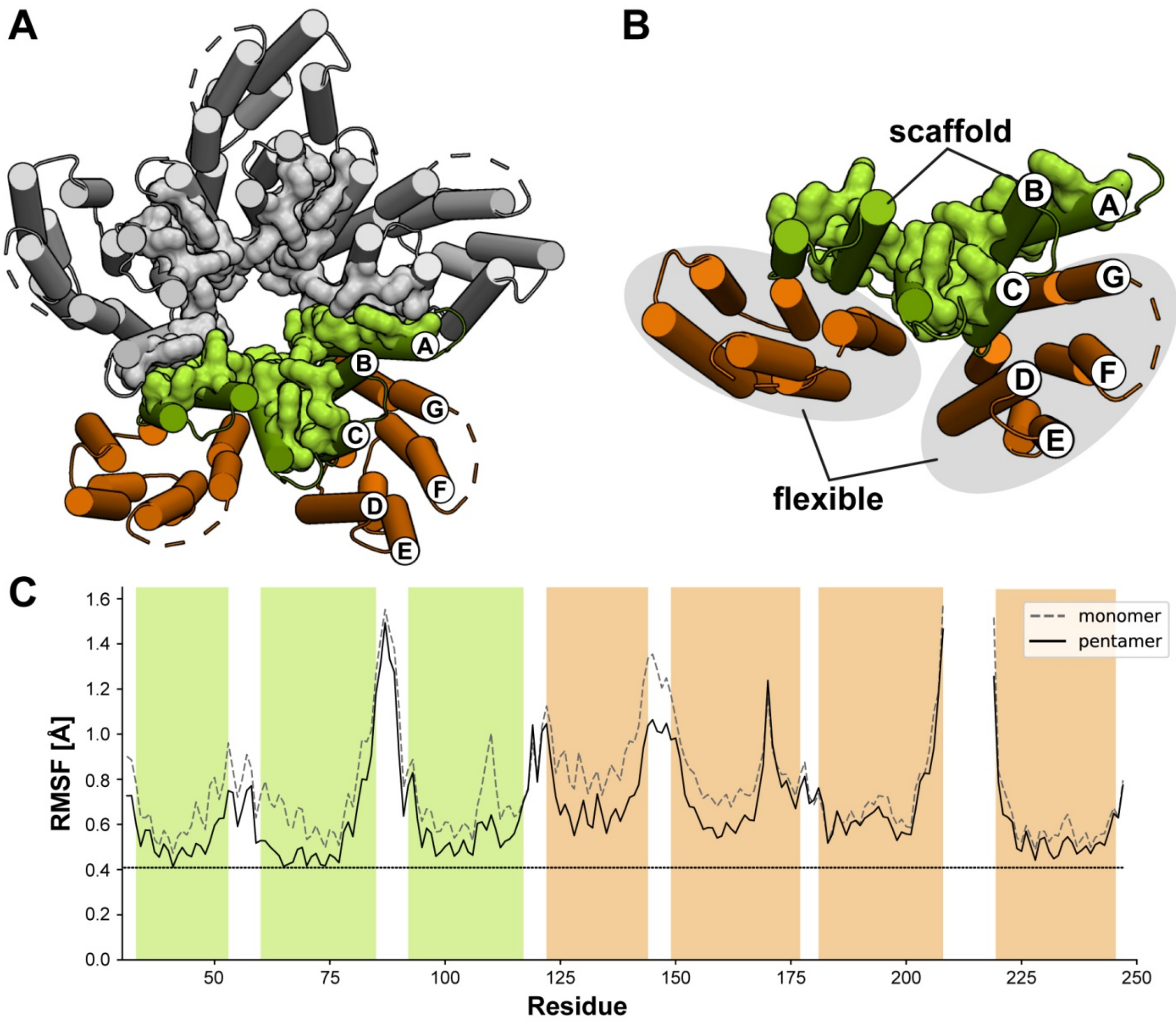

Fig. S8. Importance of the oligomeric state for functional flexibility in GPR. (A) Pentameric GPR is shown from the extracellular side with oligomerization interfaces illustrated by surfaces. Helices A, B and the N-terminal segment of helix C are involved in stabilizing the oligomerization interface (green), while the remaining helices do not contribute to these interactions (orange). (B) Closer look at two neighboring protomers. As a result of their involvement in the oligomerization, helices A, B and the N-terminal segment of helix C can act as scaffold for the functional flexibility exhibited by the C-terminal segment of helix C and helices D-G. (C) Observed Cα root mean square fluctuation (RMSF) during MD simulation of monomeric (dashed line) and pentameric (solid line) GPR. Residues corresponding to helices A-C are highlighted in green and helices D-G in orange. The lowest RMSF value is indicated with a dotted horizontal line as reference. N-terminal residues 25-30 were omitted due to high intrinsic flexibility and the E-F loop (residues 211-218) could not be built in the GPR model due to a lack of density.

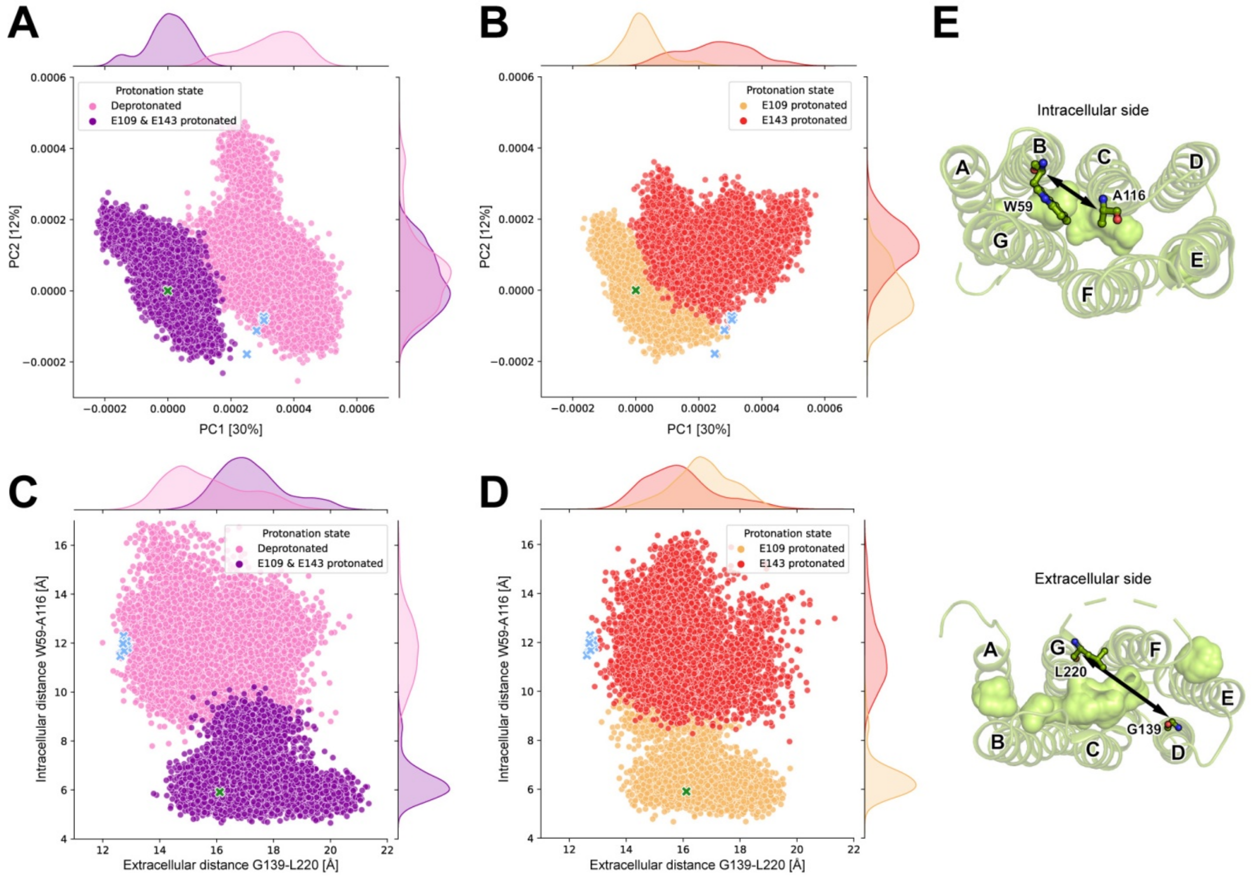

Fig. S9. Molecular dynamics simulations of lipid-embedded pentameric GPR. (A) Principle component (PC) analysis of GPR simulations with deprotonated (pink circles) or protonated (purple circles) E109 and E143. (B) PC analysis of GPR simulations with only E109 (bright orange circles) or E143 (red circles) protonated. Distances measured between the Cα-atoms of G139 and L220 on the extracellular side and between W59 and A116 on the intracellular side, are plotted for GPR with deprotonated or protonated E109 and E143 (C), and for the single protonated states (D). The marginal distributions were obtained using a kernel density estimate. Green and blue crosses indicate the GPR cryo-EM structure and the structures of BPR protomers in the pentamer, respectively. (E) Locations of residue pairs used to measure the distances in (C) and (D).

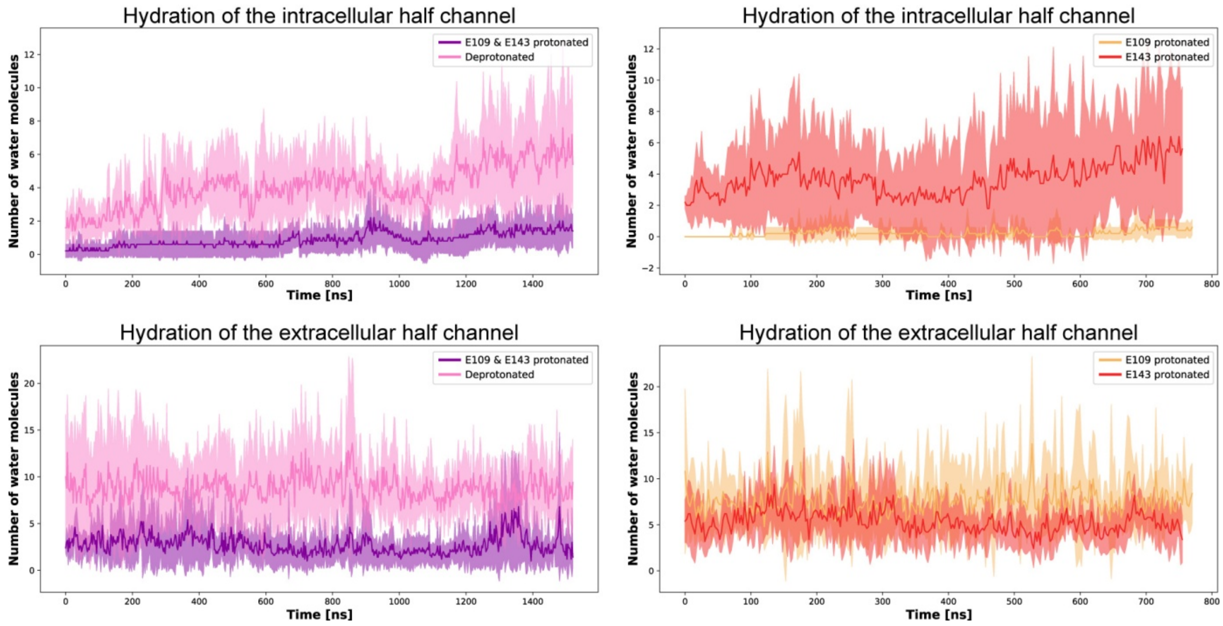

Fig. S10. Number of water molecules in the GPR half channels over the course of the MD simulations. The intracellular half of the channel was defined between the carboxyl group of E109 and the nitrogen of the Schiff base, and the extracellular half between the nitrogen of the Schiff base and the carboxyl group of E143. The central lines represent the average number of water molecule and the standard deviation is shown as shaded surfaces.

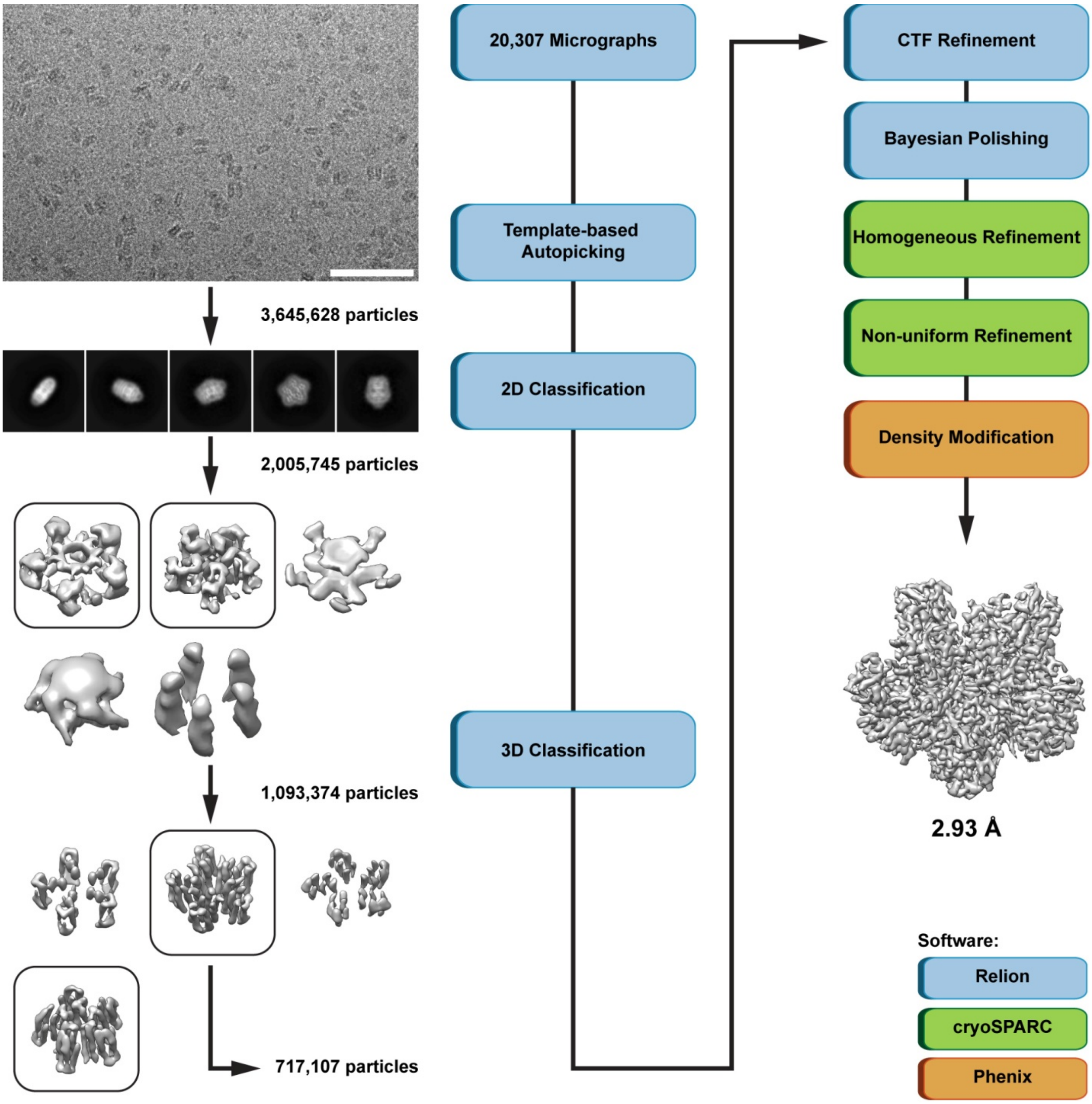

Fig. S11. Single particle cryo-EM image processing workflow. Following movie acquisition and processing, particles were picked using template-based autopicking and selected by several rounds of 2D and 3D classification. Particles were refined using CTF refinement and Bayesian polishing, followed by homogeneous and non-uniform refinement. The final map was improved by applying the Resolve Cryo-EM density modification algorithm. The scale bar in the micrograph represents 400 Å and the box size of 2D class averages is 284 Å. Software applied during the different steps are color-coded.

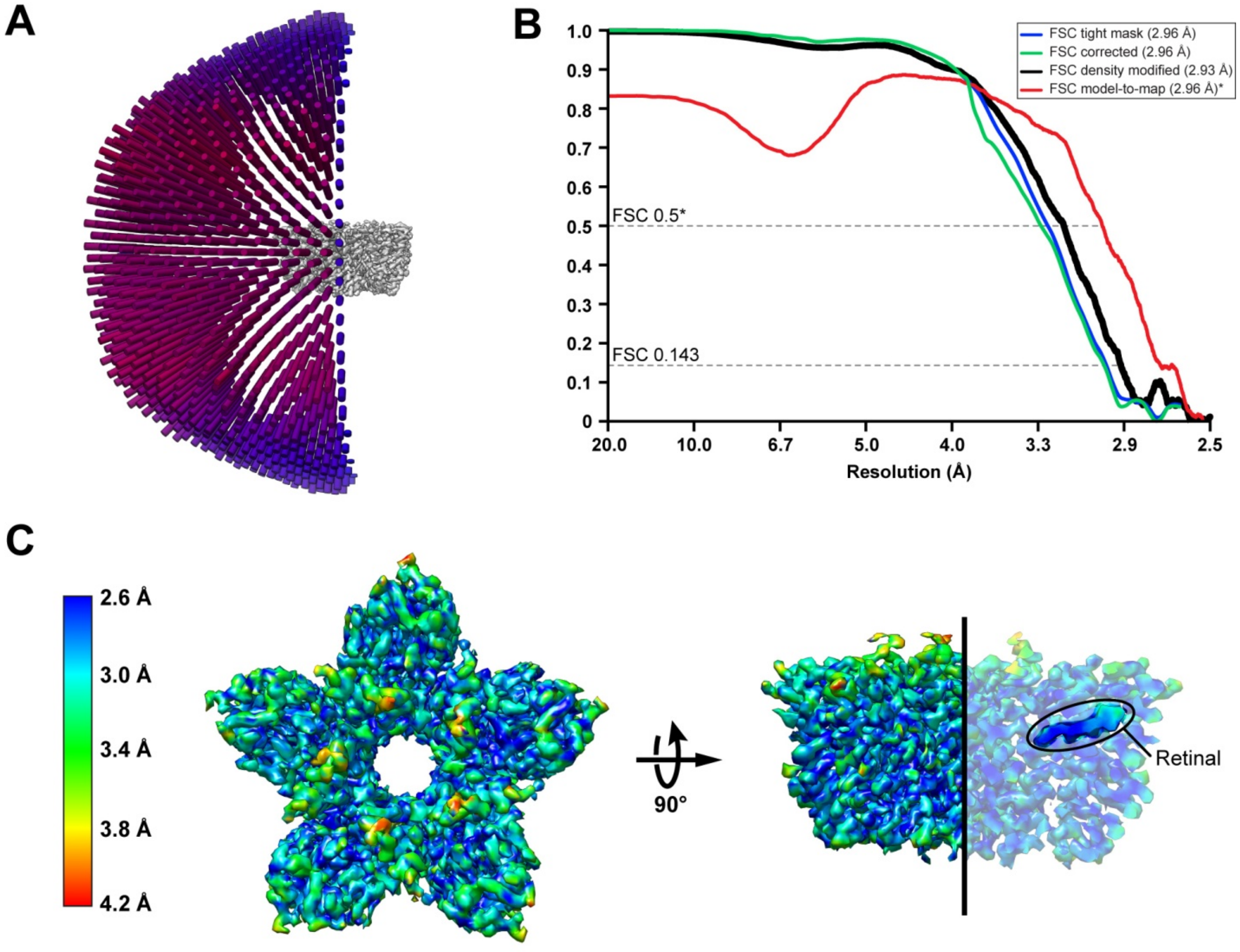

Fig. S12. Analysis of cryo-EM density map. (A) Three-dimensional representation of Euler angle distribution. Only a fifth of the complete sphere is displayed illustrating the imposed C_5 symmetry. (B) Fourier shell correlations (FSC) of experimental half-maps, density modified maps and model-to-map fit. (C) Density map colored by local resolution, calculated using MonoRes[1] implemented in cryoSPARC[2]. The side view includes a cross section illustrating the local resolution of the retinal chromophore and the surrounding binding pocket.

Table S1. Relative expression levels of GPR variants

GPR variant	Expression level (%)	Absorption maximum (nm)
wt	100	525
K58A	56	530
W59A	60	530
S180A	55	525
E246A	40	530
Y96F	71	510
E143A	72	515
Y209F	78	515
Y224F	70	510

Table S2. Cryo-EM data collection, refinement and validation statistics

**Data collection and processing**	
Microscope	Titan Krios G3
Camera	Gatan Quantum-K3
Voltage (kV)	300
Magnification	130,000x
Defocus range (μm)	-0.9 to -1.9
Movies recorded	20,307
Frames per movie	40
Total dose per movie ($e^-/Å^2$)	54.23
Pixel size (Å)	0.645
Software	Relion 3.1b
Particles refined	717,107

**Refinement**	
Software	cryoSPARC v2
Symmetry	C_5
Map resolution (Å)[a]	2.96 (2.93)[b]
Map sharpening *B*-factor ($Å^2$)	-125.4
Model composition	
Non-hydrogen atoms	8,670
Protein residues	1,085
Ligands	5

**Validation**	
Software	Phenix 1.18-3855
MolProbity score[c]	1.43
All-atom clashscore	7.91
Rotamer	
Favored (%)	97.18
Outliers (%)	0.00
Ramachandran	
Favored (%)	98.12
Outliers (%)	0.00
Ramachandran Z-score	1.34
RMSD	
Bond length (Å)	0.006
Bond angles (°)	1.156
Mean *B*-factors	
Protein ($Å^2$)	16.7
Ligands ($Å^2$)	63.7
CaBLAM outliers (%)	0.96
Cβ outliers (%)	0.00
EM-Ringer score[d]	1.85
Map CC (peak)[e]	0.67
Map CC (mask)[e]	0.81

[a] Resolution determined by Fourier shell correlation between half-maps using the gold standard cut-off of 0.143.
[b] Value in brackets represents resolution of density modified map[3].
[c] Model statistics were calculated using MolProbity[4].
[d] Calculated based on local fit of side chains to map according to Barad et al. 2015[5].
[e] Real-space correlation coefficients of model-to-map fit as described in Afonine et al. 2018[6].

References

1. Vilas, J. L. *et al.* MonoRes: automatic and accurate estimation of local resolution for electron microscopy maps. *Structure* **26**, 337–344 (2018).
2. Punjani, A., Rubinstein, J. L., Fleet, D. J. & Brubaker, M. A. cryoSPARC: algorithms for rapid unsupervised cryo-EM structure determination. *Nat. Methods* **14**, 290–296 (2017).
3. Terwilliger, T. C., Ludtke, S. J., Read, R. J., Adams, P. D. & Afonine, P. V. Improvement of cryo-EM maps by density modification. *Nat. Methods* **17**, 923–927 (2020).
4. Williams, C. J. *et al.* MolProbity: more and better reference data for improved all-atom structure validation. *Protein Sci.* **27**, 293–315 (2018).
5. Barad, B. A. *et al.* EMRinger: side chain–directed model and map validation for 3D cryo-electron microscopy. *Nat. Methods* **12**, 943–946 (2015).
6. Afonine, P. V. *et al.* New tools for the analysis and validation of cryo-EM maps and atomic models. *Acta Crystallogr. Sect. D Struct. Biol.* **74**, 814–840 (2018).
