# Supplementary material for: Cryo-EM structure and dynamics of the green-light absorbing proteorhodopsin
Source: Nat Commun. 2021 Jul 5;12:4107. doi: 10.1038/s41467-021-24429-6 (PMC8257665; doi:10.1038/s41467-021-24429-6)
Supplement: Supplementary file 4 — Description of additional supplementary files [file 41467_2021_24429_MOESM4_ESM.docx]

Description of additional supplementary files

Title: Movie S1.

Description: Morph between GPR (PDB ID: 7B03) and Hot75BPR (PDB ID: 4KLY) structures, visualizing the potential conformational changes required to invert the selective solvent accessibility to the intra- (IC) and extracellular cavities (EC). The backbone of the structure is displayed as loops (blue), important cavities along the proton translocation pathway are represented as surfaces (pink) and the proposed proton release group E143 (top), the retinal Schiff base bound to K232 (middle) and the primary proton donor E109 (bottom) are highlighted (orange).
